# PSD-95 Serine 73 phosphorylation is not required for induction of NMDA-LTD

**DOI:** 10.1038/s41598-020-58989-2

**Published:** 2020-02-06

**Authors:** Agata Nowacka, Małgorzata Borczyk, Ahmad Salamian, Tomasz Wójtowicz, Jakub Włodarczyk, Kasia Radwanska

**Affiliations:** 1 Laboratory of Molecular Basis of Behavior, Nencki Institute of Experimental Biology, Polish Academy of Sciences, Warsaw, Poland; 2 Laboratory of Cell Biophysics, Nencki Institute of Experimental Biology, Polish Academy of Sciences, Warsaw, Poland

**Keywords:** Molecular neuroscience, Membrane potential

## Abstract

PSD-95 is a major scaffolding protein of the post-synaptic density (PSD) of a glutamatergic synapse. PSD-95, via interactions with stargazin, anchors AMPA receptors at the synapse and regulates AMPAR currents. The expression of PSD-95 is regulated during synaptic plasticity. It is, however, unknown whether this regulation is required for induction of functional plasticity of glutamatergic synapses. Here, we show that NMDA-induced long-term depression of synaptic transmission (NMDA-LTD) is accompanied by downregulation of PSD-95 protein levels. Using pharmacologic and molecular manipulations, we further demonstrate that the NMDA-induced downregulation of PSD-95 depends on the activation of CaMKII and CaMKII-driven phosphorylation of PSD-95 serine 73. Surprisingly, neither CaMKII activity nor CaMKII-dependent phosphorylation of PSD-95 serine 73 are required for the expression of NMDA-LTD. These results support the hypothesis that synaptic plasticity of AMPARs may occur without dynamic regulation of PSD-95 protein levels.

## Introduction

PSD-95 is the major scaffolding protein of a glutamatergic synapse^1^ affecting its stability, activity-dependent modifications^2–5^ and functional plasticity^6–9^. PSD-95 interacts directly with NMDA receptors and with AMPA receptors through an auxiliary protein stargazin^10,11^. Interaction of PSD-95 with stargazin regulates synaptic content of AMPARs^10,12,13^. In agreement with these findings mice lacking functional PSD-95 protein have greatly enhanced hippocampal, NMDAR-dependent long-term potentiation (LTP), whereas NMDAR-dependent long-term depression (LTD) is absent^6^. Conversely, overexpression of PSD-95 occludes LTP^7,8^ and decreases the threshold for LTD induction^9^. Importantly, PSD-95 is a highly dynamic protein. Upon stimulation, PSD-95 is phosphorylated at serine 73 and transiently removed from the dendritic spine in a CaMKII-dependent manner^2^. However, inhibition of this process by mutation of serine 73 to alanine does not affect the propensity to induce LTP^2^. Sturgill *et al*. have also shown that NMDA-LTD provokes rapid destabilization of PSD-95 in the spine head^4^. It is yet unknown whether LTD-induced elimination of PSD-95 protein from the dendritic spine is necessary for the expression of LTD.

Here, using organotypic hippocampal cultures (OHC) we confirmed that PSD-95 protein levels were downregulated in the *stratum radiatum* of CA1 hippocampal field after induction of NMDA-LTD^4^. Since it has been shown that CaMKII-dependent phosphorylation of PSD-95 on serine 73 (PSD-95:Ser73) regulates the binding of PSD-95 to NMDAR^14^ and translocation of PSD-95 from activated spines^2^, we checked if CaMKII contributes to LTD-induced downregulation of PSD-95. Using pharmacological manipulations and AAV transfection approach we found that NMDA-LTD-induced downregulation of PSD-95 levels is regulated by CaMKII activity and CaMKII-driven phosphorylation of PSD-95:Ser73. Surprisingly, we also observed that neither CaMKII activity nor CaMKII-dependent phosphorylation of PSD-95:Ser73 are necessary for the expression of NMDA-LTD. Our data indicate dissociated function of CaMKII-dependent phosphorylation of PSD-95 in the regulation of molecular remodeling of synapses upon induction of NMDA-LTD and functional synaptic plasticity.

## Materials and Methods

### Organotypic hippocampal slice cultures

Organotypic hippocampal slice cultures (OHC) were prepared from 5–7 day old Wistar rats as earlier described^15^. Briefly, the hippocampi were isolated and cut into 300-μm sections with a tissue chopper (McIlwain Tissue Chopper, Ted Pella). The sections were placed in dissection medium composed of GBSS (Sigma, G9779), 0.5% D-glucose (Sigma, G8769), 100 U/ml/100 mcg/ml penicillin/streptomycin (BioShop, PST999), 10 mM HEPES (BioShop, HEP003), and incubated on ice for 30–90 min. Selected slices were transferred to a culture medium (CM): MEM (Sigma, 51412C), HBSS (Biological Industries, 02-015-1A), 0.5% D-glucose (Sigma, G9779), 100 U/ml/100 mcg/ml penicillin/streptomycin (BioShop, PST999), 2 mM L-glutamine (BioShop, GLU999), four times diluted inactivated horse serum (Gibco, 16050-122) and incubated on ice. Slices were then mounted on UV pre-sterilized membranes (Merck-Millipore, FHLC04700) and placed in inserts (Merck-Millipore, PICM03050) in 6-well culture dishes with 1 ml of culture medium per well. The interphase culture was maintained at 37 °C, 5% CO_2_ and 95% humidity for two weeks. The culture medium was changed every 3 days.

### Chemical LTD induction

NMDAR-dependent LTD was induced with 30 µM NMDA (Sigma, M3262)^4,16^. On the 14^th^ day *in vitro* (14DIV) the slices were placed in 1 ml of culture medium supplemented with 30 µM NMDA for 4 min. Then the inserts were moved back to the old CM for additional 26 minutes. In the control group, inserts were moved to fresh CM for 4 min and back to the old CM for additional 26 minutes.

### Blocking CaMKII activity

To block CaMKII activity in OHC the slices were incubated with 10 µM KN-62 (Cayman chemical, 13318) into the culture medium for 20 min before induction of NMDA-LTD. The NMDA-LTD induction mixture was supplemented with 10 µM KN-62 and the induction procedure was carried out as described above.

### Viral transduction of OHC

Adeno-associated viruses, isotype 1 and 2 (AAV1/2), were prepared from pAAV:αCaMKII-PSD95(WT)-mCherry and pAAV:αCaMKII-PSD95(S73A)-mCherry plasmids coding either wild type PSD-95 (PSD-95:WT) or a form of the protein with a point mutation of serine 73 to alanine (PSD-95:S73A) fused with fluorescent mCherry under αCaMKII promoter. OHC were transduced with AAV1/2:αCaMKII-PSD95(WT)-mCherry (viral titer: 1.35 × 10^9^/µl), AAV1/2:αCaMKII-PSD95(S73A)-mCherry (viral titer: 9.12 × 10^9^/µl), LV:αCaMKII-shRNA(PSD95)-GFP (viral titer: 1.7 × 10^7^/µl) or LV: αCaMKII-GFP (viral titer: 1.17 × 10^7^/µl) on the 7DIV. The viruses were diluted three times. 0.5 µl of the virus solution was injected into the CA1 of OHC with a glass capillary (GMBH, 7087 07) connected to a syringe.

### Immunofluorescence staining

OHC were fixed with 4% PFA (paraformaldehyde) with 4% sucrose in PBS (phosphate buffered saline) for 30 minutes at room temperature. Then they were washed 3 × 6 min with PBS and permeabilized with 0.5% Triton X-100 (Bioshop, TRX506) in PBS for 12–18 hours at 4 °C. The sections were again washed 2 × 6 min with PBS and blocked with 10% NDS (natural donkey serum) in PBS for 4 hours at 4°C. The slices were then incubated overnight at 4°C in a humid chamber in 5% NDS/0.3% Triton X-100/PBS with primary antibodies: mouse anti-PSD-95 (1:500; Merck-Millipore, MAB1598, RRID: AB_94278) and rabbit anti-mCherry (only virus-transduced slices, 1:500, Abcam, ab167453, RRID: AB_2571870). After the incubation was completed, the slices were washed 3 × 6 min with PBS. They were then incubated for 4 hours at room temperature in a humid chamber in 5% NDS/0.3% Triton X-100/PBS with secondary antibodies: anti-mouse Alexa Fluor 555 (non-transduced slices; 1: 500, Invitrogen, A31570, RRID: AB_2536180) or anti-mouse Alexa Fluor 488 (transduced slices; 1: 500, Invitrogen, A21202, RRID: AB_141607) and phalloidin conjugated to Alexa Fluor 488 to visualize the morphology of the slice (non-transduced slices, 1:2000, Invitrogen, A12379, RRID: AB_2315147) or anti-rabbit Alexa Fluor 647 (only transduced slices; 1:500; Invitrogen, A31573, RRID: AB_2536183). After incubation, the slices were washed 3 × 6 min with PBS, mounted on slides and embedded in Fluoromount G with DAPI (Invitrogen, 00-4959-52).

### OHC imaging

OHCs were imaged using Zeiss LSM800 confocal microscope. 63x planachromatic lens (Plan Apochromat 63x/1.4 Oil DIC) with oil immersion and 0.5 zoom. Lasers with wavelengths 405, 561, 488 nm were used for imaging non-transduced slices. The scans had a resolution of 1122 × 1122 px and a dimension of 78 × 78 μm. Lasers with wavelengths 488, 561, 640 nm were used for imaging virally transduced slices. The scans had a resolution of 2917 × 2917 px and a size of 202.8 × 202.8 μm. The pictures were taken in the *stratum radiatum* of the CA1 field. To calculate the frequency of cell transduction with AAV1/2 in the slices subjected to the electrophysiological recordings, the scans were taken in the *stratum pyramidale* of CA1. Three scans per slice were taken, and averaged.

### OHC image analysis

Images were analyzed using ImageJ software^17^. For non-transduced slices, the mean grey value (MGV) of fluorescence of PSD-95 fluorescent staining from the whole scans was measured. In these experiments, each data point represents one slice. To measure the number of PSD-95 clusters and the average size of a PSD-95 cluster a size threshold was used to visualize single PSD-95 clusters and their number and size were quantified with ImageJ software. Results from three scans per slice were averaged. In virus-transduced slices, only dendrites of transduced cells that expressed mCherry were analyzed. Selected dendrites were manually outlined and the MGV of PSD-95 immunofluorescence was measured. In each image, two dendrites belonging to different cells were analyzed. Here, each data point represents one cell. To calculate the percentage of cells transduced with the virus in OHC after electrophysiological recordings, cell bodies of all DAPI-stained cells and cell bodies stained with DAPI with additional mCherry expression in the cytoplasm (transduced cells) were counted. The proportion of cells transduced in the entire pool of cells measured in 3 photos per slice was averaged.

### Dissociated hippocampal cultures

Primary cell cultures of hippocampal neurons were prepared as described in Michaluk *et al*. 2011 from P0 Wistar rats^18^. On 10 DIV the cultures were transfected with pAAV:αCaMKII-PSD95(WT)-mCherry, pAAV:αCaMKII-PSD95(S73A)-mCherry and pLV:αCaMKII-shRNA (PSD95)-GFP plasmids using lipofectamine 2000 (Invitrogen, 15668-019) according to the manufacturer protocol. 1 μg of plasmid DNA was used for transfection. The cells were fixed for immunostaining on the 14 DIV.

### Immunostaining of dissociated hippocampal cultures

Cell cultures were fixed in 4% PFA in PBS solution for 8 min and washed 3 × 6 min in PBS. Next, cells were permeabilized in 0.1% Triton X-100 (Bioshop, TRX506) in PBS solution for 10 min in room temperature (RT) and blocked with 10% NDS in PBS solution for 1 hour in RT. Cells were incubated with primary antibody solution with 5% NDS, rabbit anti-mCherry antibody (1:500; Abcam, ab167453, RRID:AB_2571870) and mouse anti-PSD-95 antibody (1:500; Merck-Millipore, MAB1598, RRID:AB_94278) in PBS overnight in 4 °C. Next, the cells were washed 3 × 10 min and incubated with secondary antibody solution: 5% NDS, anti-rabbit Alexa Fluor 647 (mCherry coding plasmids; 1:500; Invitrogen, A31573, RRID:AB_2536183) and anti-mouse Alexa Fluor 488 (mCherry coding plasmids; 1:500; Invitrogen, A21202, RRID:AB_141607) or anti-mouse Alexa Fluor 555 (GFP coding plasmids; 1:500; Invitrogen; A31570; RRID:AB_2536180) in PBS for 1h in RT. Then the cells were washed 3 × 10 min and mounted on microscope slides with Fluoromount G with DAPI (Invitrogen, 00-4959-52).

### Imaging of dissociated hippocampal cultures

Preparations were visualized with Zeiss 800 confocal microscope. For imaging, 63x plan apochromat lens with oil immersion (Plan Apochromat 63x/1.4 Oil DIC) and 488 nm, 561 nm, 640 nm lasers were used. The confocal scans have a resolution of 1024 × 1024 px. Z-stacks of selected dendrites were taken (distance between scans 0.34 μm**)**. Finally, the scans were deconvoluted using AutoQuant software (Media Cybernetics). For PSD-95 silencing analysis, single scans were taken.

### Dissociated hippocampal cultures image analysis

The confocal scans were analyzed with ImageJ software^17^. For image analysis, maximal intensity z-projections of confocal scans were obtained. To assess the level of PSD-95 protein overexpression in transfected cells the Mean Grey Value (MGV) of PSD-95 fluorescence between dendrites of transfected and non-transfected cells from the same cultures were compared. To assess the level of PSD-95 silencing MGV of PSD-95 fluorescence between the cell body of and non-transfected cells from the same cultures were compared.

### Electrophysiological recordings

Recordings of excitatory postsynaptic field potentials were carried out in the *stratum radiatum* of the CA1 field of OHC at the age of 9–14 DIV. All measurements were performed in a submerged chamber in recirculated artificial cerebrospinal fluid (ACSF) with a composition of 126 mM NaCl, 2.5 mM KCl, 1.25 mM NaH_2_PO_4_, 2.5 mM CaCl_2_, 1.3 mM MgCl_2_, 26 mM NaHCO_3_, 10 mM D-glucose, oxygenated carbogen (95% O_2_, 5% CO_2_) at room temperature. Extracellular excitatory postsynaptic field potentials (fEPSP) were recorded using MultiClamp 700B software (Molecular Devices, California, USA) and Clampex 10.0. Synaptic responses were induced with a bipolar stimulating electrode (FHC, CBARC75) stimulating Schaffer collaterals in CA3 of OHC. The stimulating electrode was connected to a stimulator (A.M.P.I Iso-Flex). Synaptic responses were recorded in the *stratum radiatum* of CA1 with a chlorinated silver wire placed in an ACSF filled glass electrode (resistance 1–3 MΩ). The glass electrodes were pulled from borosilicate glass (WPI, 1B120F-4) using a micropipette puller (Sutter Instruments, P-1000). The intensity of stimulation was adjusted to evoke 50% of the maximum fEPSP. The stimulation pulse was delivered every 10 seconds. During the experiments, 20 min of baseline fEPSP was recorded. In the case of control recordings, slices were perfused with ACSF only and fEPSP were recorded for a total of 80 minutes. During NMDA-LTD induction, after 20 min baseline recording, slices were perfused with 30 μM NMDA for 4 minutes. Then the signals were recorded for 56 min in ACSF. In the case of recordings with CaMKII inhibitor, KN-62 at a concentration of 10 μM was added to the culture medium 20 min before the recording. The entire recording took place in ACSF with 10 μM KN-62. During NMDA-LTD induction, the slice was perfused with 30 μM NMDA with the addition of 10 μM KN-62. In the control group, the recording and induction were carried out in the presence of an equal amount of DMSO solvent. In the case of the recordings with tatCN21, a 10 min baseline recording (ACSF), followed by 15 min recording of baseline [tatCN21(5 µM) or tatCtrl (5 µM)]. Next, the slices were perfused for 4 min with 30 μM NMDA with 5 µM tatCN21/tatCtrl. The rest of the recording was performed in ACSF only. In the case of the recordings with an open-channel NMDAR blocker, after 20 min of baseline recording the slices were perfused with 50 μM MK-801 for 20 minutes (Tocris, 0924). Then NMDA-LTD was induced as described above.

### Analysis of electrophysiological data

fEPSPs were analyzed with Clampfit software (Molecular Devices, California, USA). For all measurements, the slope of fEPSP expressed in mV/ms was measured. The obtained results were then normalized to the average of the first 20 min of baseline and multiplied by 100 to achieve a relative percentage value. Statistical data were analyzed with GraphPad Prism 6 software (La Jolla California, USA). In the described experiments, one data point (n) is a slice.

### Statistical analysis

Description of the used statistical tests and the information on the size of experimental groups can be found in the descriptions under the figures. The difference between the groups was considered significant for P < 0.05.

## Results

### NMDA induces long-term synaptic depression through activation of synaptic NMDA receptors

We used NMDA-induced chemical LTD (NMDA-LTD) protocol (30 μM NMDA for 4 minutes) in the organotypic cell cultures (OHC) to produce global depotentiation of synaptic transmission^4,16,19–21^. OHC was chosen as a simplified model of the hippocampal formation that preserves most of the synaptic organization of the hippocampal area CA1^22^. fEPSPs were recorded in the CA1 while stimulating the Schaffer collaterals in the CA3 (Fig. S1a). We decided to analyze the slope of fEPSP because OHCs are highly excitable^22^ and the fEPSP measured in CA1 is often contaminated by the population spike.

**Figure 1:**
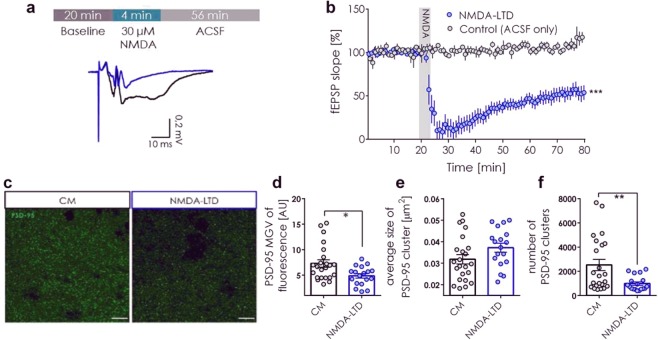
NMDA stimulation of OHC induced depression of synaptic transmission and downregulated PSD-95 protein. (**a**) The scheme of the experiment. During electrophysiology recordings fEPSPs were recorded in the CA1 of OHCs while stimulating the Schaffer collaterals in the CA3. 30 μM NMDA was administered for 4 min to induce NMDA-LTD. Exemplary trace of fEPSP before (black) and after (blue) NMDA-LTD induction. **(b)** The slope of fEPSP was stable during control recordings (grey) (slices: Control (ACSF only) = 5; the mean value of fEPSP slope from the baseline and last 30 min of recording was compared; Shapiro-Wilk normality test and paired t-test; t(4) = 1.959, p = 0.1217). Slope of fEPSP decreased by 50% after NMDA (30 μM) administration (slice: LTD = 7; the mean value of fEPSP slope from the baseline and last 30 min of recording was compared; Shapiro-Wilk normality test and paired t-test; t(6) = 7.756, p = 0.0002). **(c)** Exemplary confocal, single scans of PSD-95 immunostaining taken in the *startum radiatum* of the CA1. Scale bar is 10 μm. (**d**) PSD-95 mean grey value of photographs decreased 30 min after NMDA-LTD induction (n = slice: 23 CM, 18 NMDA-LTD; D’Agostino-Pearson normality test and unpaired t-test; t(39) = 2.683, p = 0.0107). (**e**) NMDA-LTD induction had no effect on average size of a PSD-95 cluster (n = slice: 23 CM, 18 NMDA-LTD; D’Agostino-Pearson normality test and unpaired t-test; t(39) = 1.744, p = 0.0890). (**f**) The number of PSD-95 clusters decreased after NMDA-LTD induction (n = slice: 23 CM, 18 NMDA-LTD; D’Agostino-Pearson normality test and unpaired t-test; t(39) = 2.819, p = 0.0075).

First, we performed the control recordings in ACSF only to test if the OHCs give stable responses during the recording period (Fig. 1a,b). No significant change of fEPSP slope was observed during the 60-minute recording. To induce LTD, after 20 minutes of the baseline recordings, OHCs were perfused with NMDA for 4 minutes and the recording was continued in ACSF only for additional 56 minutes. Upon NMDA administration the slope of fEPSP dropped and stabilized at the level of 50% of the baseline slope (Fig. 1a,b).

NMDA-LTD is a strong stimulation protocol, where the entire tissue is flooded with a neurostimulant^16^. This produces the risk of unspecific LTD induction through extrasynaptic NMDARs^23^. To assess the contribution of extrasynaptic NMDARs to NMDA-LTD, we performed field potential recordings in the presence of MK-801 (50 μM), the NMDAR open-channel blocker (Fig. S1). MK-801 binds inside of the NMDAR ion channel when it is open and prevents current flow^24,25^. Thus, MK-801 can bind only to synaptic NMDARs that have been opened upon glutamate release from presynaptic terminals by the stimulating pulses. MK-801 administration before NMDA-LTD induction (Fig. S1c) completely blocked synaptic depression (Fig. S1). These results indicate that NMDA-LTD induction protocol is specific for synaptic NMDARs.

### The effect of NMDA-LTD on PSD-95 levels in the area CA1 of OHC

PSD-95 levels were assessed in the area CA1 of OHCs after induction of NMDA-LTD^16^, using immunofluorescence staining for PSD-95 protein and confocal imaging (Fig. 1c). OHCs were stimulated with NMDA (30 μM), or culture medium as control, for 4 minutes and fixed 26 minutes later. We chose this time point because NMDA-LTD was shown to decrease the number of tethered PSD-95 molecules in the dendritic spine head after 30 minutes^4^. It was, however, unclear if the loss of PSD-95 is related to the increased protein trafficking from the spine or global decrease in its levels. We found that 30 minutes after induction of NMDA-LTD PSD-95 protein level in the *stratum radiatum* was lower than in the control group (Fig. 1c). In particular, the number of PSD-95 clusters decreased after NMDA-LTD, while the average size of a cluster was not affected (Fig. 1d,e).

### The role of CaMKII in the regulation of NMDA-LTD and expression of PSD-95

**Figure 2:**
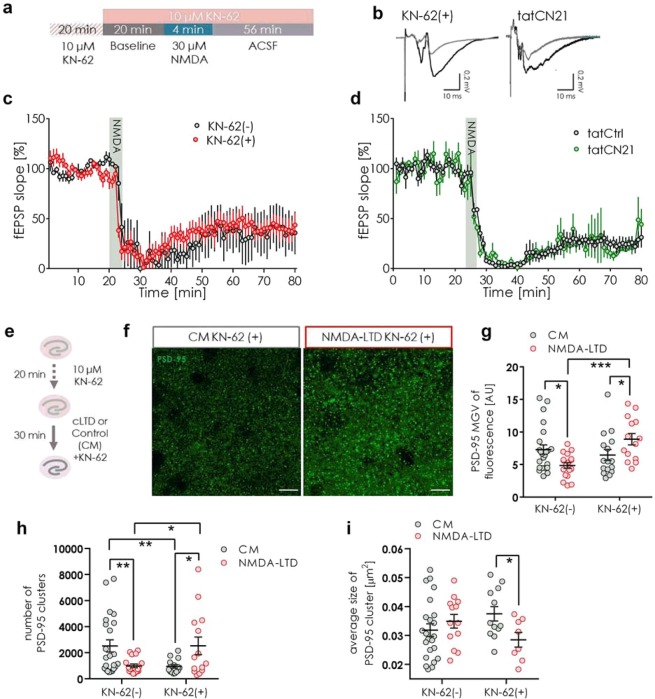
CaMKII activity does not regulate synaptic transmission during induction of NMDA-LTD but controls down-regulation of PSD-95. (**a**) Scheme of experiment. (**b**) Exemplary traces of fEPSPs before (black) and after (grey) NMDA-LTD induction for OHCs treated with KN-62 or tatCN21. (**c**) Synaptic depression was induced by NMDA in the presence of KN-62 (mean of fEPSP slope from last 30 minutes of recording was compared between groups; slice: KN-62(+) = 7, KN-62(−) = 5; Shapiro-Wilk normality test and t-test; t(10) = 0.2365, p = 0.8178). (**d**) Synaptic depression was induced by NMDA in the presence of tatCN21 (mean of fEPSP slope from last 30 minutes of recording was compared between groups; slice: tatCtrl = 5, tatCN21 = 4; Shapiro-Wilk normality test and t-test; t(7) = 0.035, p = 0.832). (**e**) Scheme of experiment to analyze PSD-95 expression during LTP. (**f**) Exemplary confocal single scans of immunostaining for PSD-95 taken in the *stratum radiatum* of CA1. Scale bar is 10 μm. (**g**) If CaMKII activity is blocked during LTD, PSD-95 level is upregulated (slice: CM KN-62 (+) = 16, NMDA-LTD KN-62 (+) = 14; Data for CM and NMDA-LTD are the same as on Fig. 1; Two-way ANOVA Fisher’s LSD test, *p < 0.05, **p < 0.01, ***p < 0.001). (**h**) With CaMKII activity blocked the number of PSD-95 clusters increased after NMDA-LTD induction (slice: CM KN-62 (+) = 16, NMDA-LTD KN-62 (+) = 14; Data for CM and NMDA-LTD groups is the same as on Fig. 1k; Two-way ANOVA Fisher’s LSD test, *p < 0.05, **p < 0.01, ***p < 0.001). (**j**) Blocking CaMKII activity resulted in a decrease in average PSD-95 cluster size after NMDA-LTD induction (slice: CM KN-62 (+) = 16, NMDA-LTD KN-62 (+) = 14; Data for CM and NMDA-LTD groups is the same as on Fig. 1l; Two-way ANOVA Fisher’s LSD test, *p < 0.05, **p < 0.01, ***p < 0.001).

It was previously shown that upon stimulation of NMDAR CaMKII-dependent phosphorylation of PSD-95 at serine 73 causes dissociation of PSD-95 from NR2A subunits of NMDARs^14^, followed by elimination of PSD-95 from dendritic spine and termination of dendritic spine growth^2^. Here, we wanted to check if CaMKII mediates NMDA-LTD and NMDA-LTD-induced downregulation of PSD-95 protein. To this end we blocked CaMKII activity with a competitive inhibitor of the kinase, KN-62 (10 μM), 20 minutes before and during induction of NMDA-LTD (Fig. 2a). Next, we assessed the extent of NMDA-LTD and the levels of PSD-95 protein (Fig. 2f). NMDA-LTD was induced in both experimental groups (Fig. 2b,c). We repeated the experiment using a peptide inhibitor of CaMKII activity, tatCN21^24^. The presence of tatCN21 did not prevent the induction of NMDA-LTD (Fig. 2d). Thus, blocking CaMKII activity did not affect synaptic transmission as compared to the control recordings, suggesting that NMDA-LTD does not rely on the activation of CaMKII.

Surprisingly, when CaMKII activity was blocked the PSD-95 level was increased after NMDA-LTD induction indicating that CaMKII activity regulates PSD-95 downregulation (Fig. 2e–g). Moreover, the number of PSD-95 clusters increased after NMDA-LTD with CaMKII activity blocked (Fig. 2h) and their average size was smaller (Fig. 2i), suggesting the generation of small PSD-95 spots.

### The role of phosphorylation of PSD-95 at serine 73 in NMDA-LTD and expression of PSD-95

**Figure 3:**
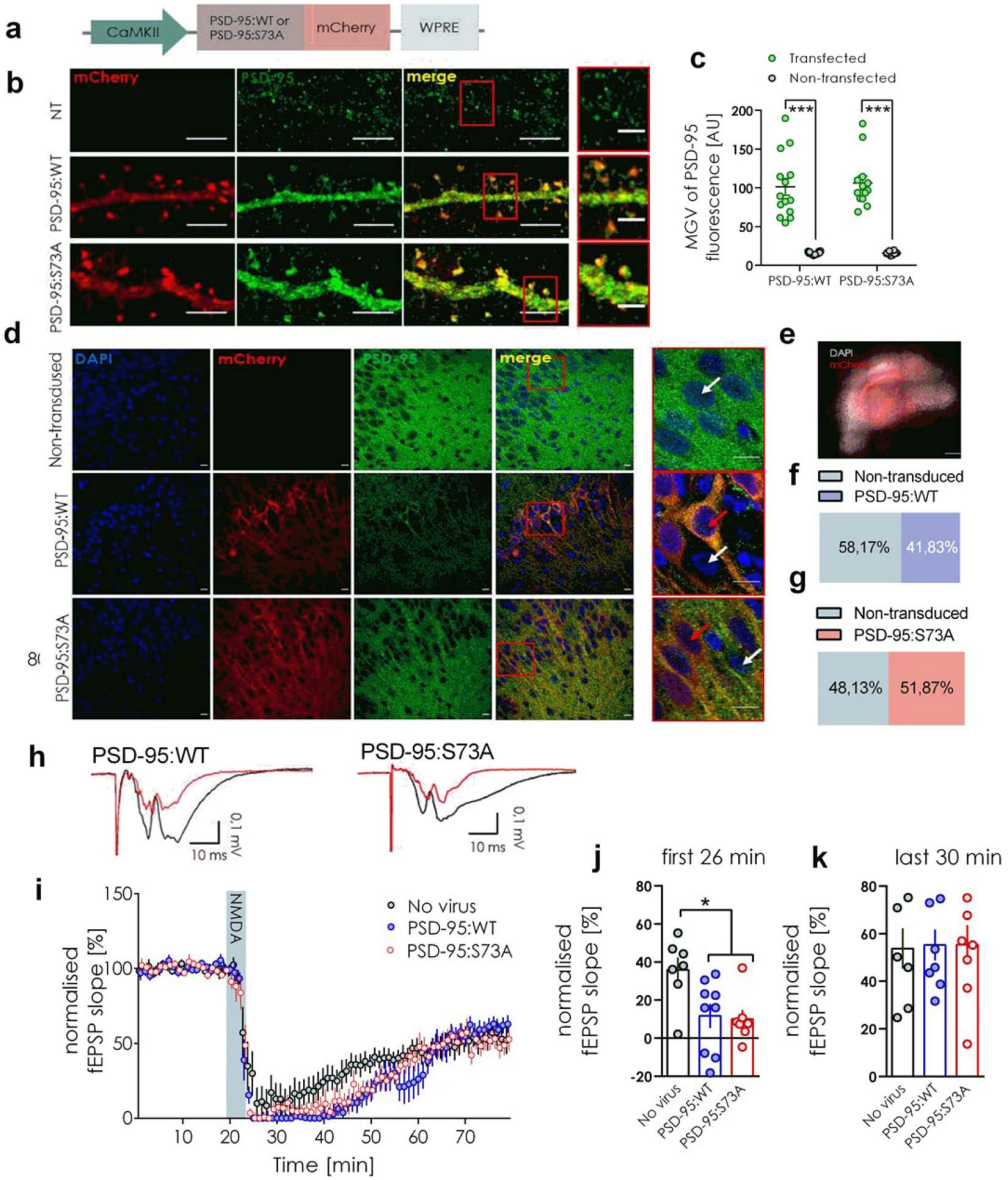
CaMKII-dependent phosphorylation of PSD-95 Ser73 does not regulate synaptic transmission during NMDA-LTD induction. (**a**) Scheme of pAAV:αCaMKII-PSD95(WT)-mCherry and pAAV:αCaMKII-PSD95(S73A)-mCherry plasmids. (**b**) Confocal z-projections of dissociated hippocampal culture dendrites transfected with the plasmids. Scale bar is 5μm and 2 μm for the magnifications. (**c**) The MGV of PSD-95 fluorescence in transfected dendrites was significantly higher than in non-transfected dendrites from the same slides (cells: PSD-95:WT transfected = 14, PSD-95:WT non-transfected = 12, PSD-95:S73A transfected = 13, PSD-95 non-transfected = 10; Two-way ANOVA; ***p < 0.001). (**d**) Exemplary confocal single scans of AAV transduced OHCs. Scale bar is 10 μm and 5 μm for the magnifications. Red arrows indicate transduced cells while white arrows indicate non-transduced cells. (**e**) Exemplary photo of an AAV tranduced OHC. (**f**,**g**) percent of AAV transduced cells in OHCs. (**h**) Exemplary traces of fEPSP before (black) and after (red) NMDA-LTD induction. (**i**) Slope of fEPSP of PSD-95:WT, or PSD-95:73A-expressing OHCs and non-transduced OHCs during NMDA-LTD (slice: No virus = 7, PSD-95:WT = 8, PSD-95:S73A = 8). (**j**) The relative slope of fEPSP during the first 26 min from induction was lower in PSD-95:WT and PSD-95:S73A-expressing OHCs as compared to the controls (mean fEPSP slope during the first 26 minutes from LTD induction was compared between groups; unpaired t-test, t(13) = 2.252, p = 0.0423). (**k**) During the last 30 min of recording no difference in fEPSP slope was observed between the experimental groups (mean fEPSP slope during the first 26 minutes from induction was compared between groups; one-way ANOVA, F(2, 20) = 5.370, P = 0.013, *P < 0.05).

A number of posttranslational modifications of PSD-95 affect its synaptic localization and expression^25^. In particular, upon stimulation with NMDA, PSD-95 is phosphorylated on serine 73^14^ and transiently removed from the dendritic spine in CaMKII-dependent manner^2^. Therefore, we examined if CaMKII-dependent downregulation of PSD-95 after NMDA-LTD induction is regulated by phosphorylation of Serine 73. We induced overexpression of wild-type (PSD-95:WT) and phosphorylation-deficient (PSD-95:S73A) PSD-95 in dissociated hippocampal culture (Fig. 3a). The plasmids caused significant overexpression of exogenous PSD-95 (Fig. 3b,c), therefore we used them for production of AAV and transduction of CA1 neurons in OHC (Fig. 3d,e). In the area CA1 of OHCs, the transduction rate yielded 41% of PSD-95:WT (Fig. 3f) and 51% of PSD-95:S73A transduced cells (Fig. 3g). We performed field potential recordings in the CA1 of transduced OHCs during NMDA-LTD induction. The relative slopes of fEPSPs of the slices transduced with PSD-95:WT or PSD-95:S73A were lower during the first 26 min after LTD induction than in non-transduced OHCs but then stabilized at the same level (Fig. 3i–k). This suggests that increased levels of PSD-95 could slow down the recovery of response after NMDA-LTD induction. The expression of phosphorylation-deficient PSD-95:S73A did no effect fEPSP slope as compared to PSD-95:WT group (Fig. 3i–k). This suggests that CaMKII-driven phosphorylation of PSD-95 Ser73 does not affect synaptic transmission during NMDA-LTD.

The lack of effect of PSD-95:S73A mutation on the fEPSP slope could be due to the fact that only a fraction of the cells expressed mutated protein. Thus we performed a technical control and tested if the virus transduction method in OHC is sufficient to induce global changes in the synaptic transmission in the area CA1. To this end we used viruses encoding shRNA targeted against PSD-95 mRNA (CaMKII_shRNA_PSD95_eGFP)^26^, as the acute knock-down of PSD-95 with shPSD-95 reduces the magnitude of LTD and the strength of the basal synaptic transmission^27^. The shRNA downregulated the levels of PSD-95 protein (Supplementary Fig. 2a,b). When the virus encoding shRNA was applied to OHCs we were unable to record fEPSPs in the shRNA:PSD-95 expressing slices, while the fiber volley was still observed proving successfully stimulation of the Schaffer collaterals. Moreover, fEPSPs in the control GFP-transduced OHCs were normal (Supplementary Fig. 2c). Thus fEPSPs in OHC can be efficiently manipulated by the local injection of the virus.

**Figure 4:**
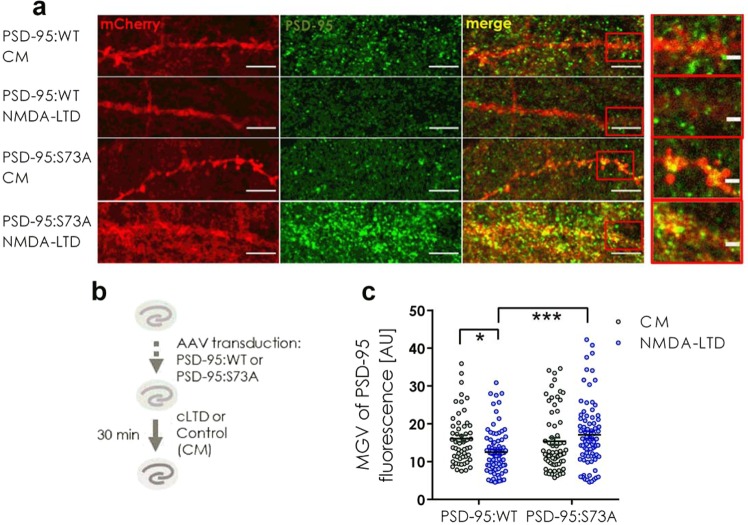
PSD-95 downregulation is regulated by CaMKII-dependent phosphorylation of PSD-95 Ser73. (**a**) Representative confocal scans of dendrites expressing PSD-95:WT and PSD-95:S73A immunostained for PSD-95 and mCherry. Scale bar is 5 μm and 1 μm for the magnification. (**b**) Scheme of experiment. (**c)** In PSD-95:WT expressing cells PSD-95 was downregulated after NMDA-LTD induction. In PSD-95:S73A expressing cells this downregulation was blocked (cell: PSD-95:WT CM = 55, PSD-95:WT NMDA-LTD = 80, PSD-95:S73A CM = 64, PSD-95:S73A NMDA-LTD = 81; Two-way ANOVA with Tukey’s post-hoc; *p < 0.05, ***p < 0.001).

Then, we induced NMDA-LTD for 30 minutes (Fig. 4b), immunostained OHCs for PSD-95 and visualized transduced cells with confocal microscopy (Fig. 4a). Consistently with the previous results, the total PSD-95 level was downregulated 30 minutes after NMDA-LTD induction in PSD-95:WT expressing dendrites (Fig. 4c). On the other hand expression of phosphorylation-deficient PSD-95:S73A prevented PSD-95 downregulation during NMDA-LTD (Fig. 4c). These results show that PSD-95 downregulation upon NMDA-LTD induction is regulated by CaMKII-dependent phosphorylation of PSD-95 at serine 73.

## Discussion

In this study, we analyzed the changes in PSD-95 protein levels in the area CA1 after NMDA-LTD induction using the OHC model. We found that 30 min after NMDA-LTD induction PSD-95 is downregulated. Moreover, we observed that blocking CaMKII activity with KN-62 inhibitor prevented NMDA-LTD-induced downregulation of PSD-95. Using overexpression of phosphorylation deficient PSD-95:S73A we found that PSD-95 downregulation is regulated by CaMKII-driven phosphorylation of PSD-95 on serine 73. Surprisingly, the field potential recordings have shown that synaptic transmission during NMDA-LTD is regulated neither by CaMKII activity nor by PSD-95:Ser73 phosphorylation. In conclusion, we propose that CaMKII-driven phosphorylation of PSD-95:Ser73 play distinct roles in the regulation of PSD-95 protein levels and functional synaptic plasticity.

### NMDA-LTD induces synaptic depression through synaptic NMDARs

To analyze the changes in PSD-95 levels globally in the whole *stratum radiatum* layer of OHC we needed to use a protocol that would elicit polysynaptic LTD. To that extent, we used the well-established NMDA-LTD induction protocol^4,16,21,28^. In our model NMDA application depressed synaptic transmission in the CA1 by 50%. A brief application of NMDA has been shown to produce postsynaptic LTD in CA1 synapses that resemble homosynaptic LTD elicited by 1 Hz electrical stimulation^16^. However, NMDA-LTD induction protocol is strong since the entire tissue is flooded with a neurostimulant. It has been shown that even 1/3 of the existing NMDARs are found extrasynaptically^29^ and they can be activated during glutamate spill-over^30–33^. To rule out the possibility of non-specific LTD induction through extrasynaptic NMDARs we performed field potential recordings during NMDA-LTD induction on OHCs whose synaptic NMDARs were blocked with MK-801, an open NMDAR channel blocker^34,35^. With synaptic NMDARs blocked, we were unable to produce synaptic depression proving that our induction protocol is specific for synaptic NMDARs.

### PSD-95 downregulation upon NMDA-LTD induction

It has been previously shown that upon brief stimulation of dendritic spines with NMDA a rapid loss of PSD-95 protein from dendritic spine heads occurs^4^. Upon NMDA-LTD induction 60% of PSD-95 was removed from the spine head and this decrease was persistent and caused by the destabilization of PSD-95 localized in the spines^4^. The decrease of synaptic PSD-95 levels could be due to dissociation of PSD-95 protein from the spine head or by the dissociation and degradation of the protein. Our results are consistent with the second hypothesis. Here, we show that PSD-95 protein was downregulated in the *stratum radiatum* layer of CA1 pyramidal neurons 30 min after NMDA-LTD induction. We observed a decrease in the number of PSD-95 clusters but the average size of the cluster was not changed suggesting that some PSD-95 clusters disappeared. In favor of the protein degradation hypothesis, it has been also shown that upon NMDARs activation, PSD-95 is ubiquitinated by the E3 ligase Mdm2 and then removed from synaptic sites by the proteasome degradation pathway^36^.

Although the analysis of PSD-95 immunostaining does not univocally indicates that NMDA-LTD induces changes of the synaptic proteins, the broad literature shows that PSD-95 is highly enriched in the post-synaptic density (PSD)^37–40^, therefore NMDA-induced changes of PSD-95 levels most probably indicate changes of the synaptic protein. We therefore hypothesize that PSD-95 downregulation after NMDA-LTD induction plays a role in the regulation of the synaptic content of AMPARs. AMPARs are localized to synapses through the binding of the first two PDZ domains of PSD-95 to the AMPARs auxillary protein stargazin^10^. The exchange of synaptic and extrasynaptic AMPARs by lateral diffusion depends mostly on the interaction of PSD-95 with stargazin and the disruption of this interaction increases AMPAR surface diffusion and counteracts AMPARs accumulation at the synapse. AMPARs and stargazin diffuse on the surface membrane in a complex and thus synaptic PSD-95 act as trapping slots for these complexes^12^. The observed downregulation of PSD-95 after NMDA-LTD induction could be, thus, a mechanism of decreasing the number of synaptic trapping slots regulating the synaptic content of AMPARs. This hypothesis is, however, opposed by the observation that the accumulation of PSD-95 does not preclude LTD. Thus, it is plausible that synaptic AMPAR content during LTD is regulated rather by PSD-95-stargazin interactions and stargazin phosphorylation status^41,42^, then by the number of PSD-95 synaptic slots.

### CaMKII role in PSD-95 downregulation

PSD-95 binding to NR2A subunit of NMDAR can be regulated by CaMKII phosphorylation of PSD-95 at serine 73, so that the phosphorylation results in the detachment of PSD-95 from NR2A^14^. The role of CaMKII in the regulation of LTP is well-established^43–45^, however, little is known about the role of the kinase in LTD. One report states that in the cultured hippocampal neurons expression of a phosphomimetic CaMKII-T286D produced synaptic depression through a long-term depression (LTD)-like process^46^. We, thus, hypothesized that the observed downregulation of PSD-95 after NMDA-LTD induction could be regulated by CaMKII activity. We found that blocking CaMKII activity with KN-62 inhibitor prevented the downregulation of PSD-95 protein after NMDA-LTD induction. In fact, the expression of PSD-95 was elevated after NMDA-LTD induction with CaMKII activity blocked. These results support the role of CaMKII in the regulation of PSD-95 levels during LTD and also reveal that PSD-95 protein is not only eliminated but also produced during NMDA-LTD, the latter process being likely CaMKII-independent. Moreover, we found that this regulation relies on CaMKII-dependent phosphorylation of PSD-95. In the cells expressing the phosphorylation deficient PSD-95:S73A, PSD-95 protein accumulated during LTD. It is, therefore, plausible that upon NMDARs activation PSD-95 is phosphorylated by CaMKII on serine 73 and this promotes the elimination of PSD-95 from dendritic spine and downregulation of PSD-95. Furthermore, as Steiner *et al*.^2^, we could not block synaptic plasticity by the expression of a phosphorylation-deficient form of PSD-95.

### The effect of CaMKII-driven phosphorylation of PSD-95 Ser73 on synaptic transmission

It has been shown that blocking CaMKII activity with a specific tatCN21 inhibitor blocked electrically-induced LTD in the acute slices^47^. We thus checked if blocking CaMKII activity in OHCs would also affect synaptic transmission during NMDA-LTD induction. Using CaMKII inhibitors, KN-62 and tatCN21, we found no change in fEPSP slope as compared with the control groups. These results suggest that CaMKII activity does not regulate synaptic transmission in our experimental model. The discrepancies between our results and those of Coultrap *et al*.^47^ may arise from the differences in the induction protocol. Here, we used chemical LTD induction, which is stronger than electrical induction^16^. Morover, CaMKII is not the only kinase involved in LTD. For example, PSD-95 has been shown to be phosphorylated on threonine 19 by glycogen synthase kinase-3β (GSK-β). Impaired phosphorylation of PSD-95:Thr19 impaired LTD induction^48^. The phosphorylation of threonine 321 in stargazin by protein kinase A (PKA) has been shown to disrupt the interaction between PSD-95 and stargazin and synaptic localization of AMPARs^49^. It has also been shown that the phosphorylation of the C-tail of stargazin affects its binding to PSD-95 by regulating the length of the C-tail and its access to PDZ domains in the cytoplasm of the spine^42^. Thus it is possible that the lack of the effect on NMDA-LTD could be due to compensatory mechanisms that are operating when CaMKII activity is blocked.

We performed field potential recording on OHCs expressing PSD-95:WT and PSD-95:S73A. PSD-95 overexpression has been shown to mimic LTP and enhance LTD^7,8^. On the other hand PSD-95:S73A overexpression has been shown to not affect LTP induction as compared to PSD-95:WT expressing cells^2^. We thus hypothesized that expression of PSD-95:WT in OHCs would facilitate NMDA-LTD induction and expression of PSD-95:S73A would block it since it impairs PSD-95 downregulation. Surprisingly, we observed no change in the level of induced synaptic depression in PSD-95:WT and PSD-95:S73A transduced OHCs as compared to control recordings. In PSD-95:WT expressing OHCs the recovery of fEPSP after NMDA-LTD induction was slightly impaired however, no change in the final synaptic depression level was found. These results show that although CaMKII-driven phosphorylation of PSD-95:Ser73 regulates the level of PSD-95 protein it does not affect synaptic plasticity in our model. This suggests a distinct role of CaMKII and PSD-95 Ser73 phosphorylation in regulating protein content and functional synaptic plasticity.

## Conclusions

We have examined the changes in PSD-95 levels during the induction of NMDA-LTD in the *stratum radiatum* of the CA1 field of OHCs. We found that PSD-95 is downregulated after NMDA-LTD induction and this is regulated by CaMKII-driven phosphorylation of PSD-95 Ser73. Surprisingly, neither CaMKII activity nor CaMKII-dependnet PSD-95 Ser73 phosphorylation affect synaptic transmission during NMDA-LTD induction suggesting distinct mechanisms that regulate PSD-95 protein content and LTD.

## Supplementary information

Supplementary Figures.

## Data Availability

The original data will be avaliable from KR upon request.
