# Supplementary material for: PSD-95 Serine 73 phosphorylation is not required for induction of NMDA-LTD
Source: Sci Rep. 2020 Feb 6;10:2054. doi: 10.1038/s41598-020-58989-2 (PMC7005143; doi:10.1038/s41598-020-58989-2)
Supplement: Supplementary file 1 — Supplementary Figures. [file 41598_2020_58989_MOESM1_ESM.pdf]

PSD-95 Serine 73 phosphorylation is not required for induction of NMDA-LTD

Running title: Regulation of PSD-95 during LTD

Agata Nowacka[1], Małgorzata Borczyk[1], Ahmad Salamian[1], Tomasz Wójtowicz[2], Jakub Włodarczyk[2], Kasia Radwanska[1*]

[1]Laboratory of Molecular Basis of Behavior, Nencki Institute of Experimental Biology, Polish Academy of Sciences, Warsaw, Poland

[2]Laboratory of Cell Biophysics, Nencki Institute of Experimental Biology, Polish Academy of Sciences, Warsaw, Poland

*__Correspondence:__

Kasia Radwanska, Ph.D.,

k.radwanska@nencki.gov.pl; tel: +48501736942

Key words: PSD-95, CaMKII, synaptic plasticity, LTD

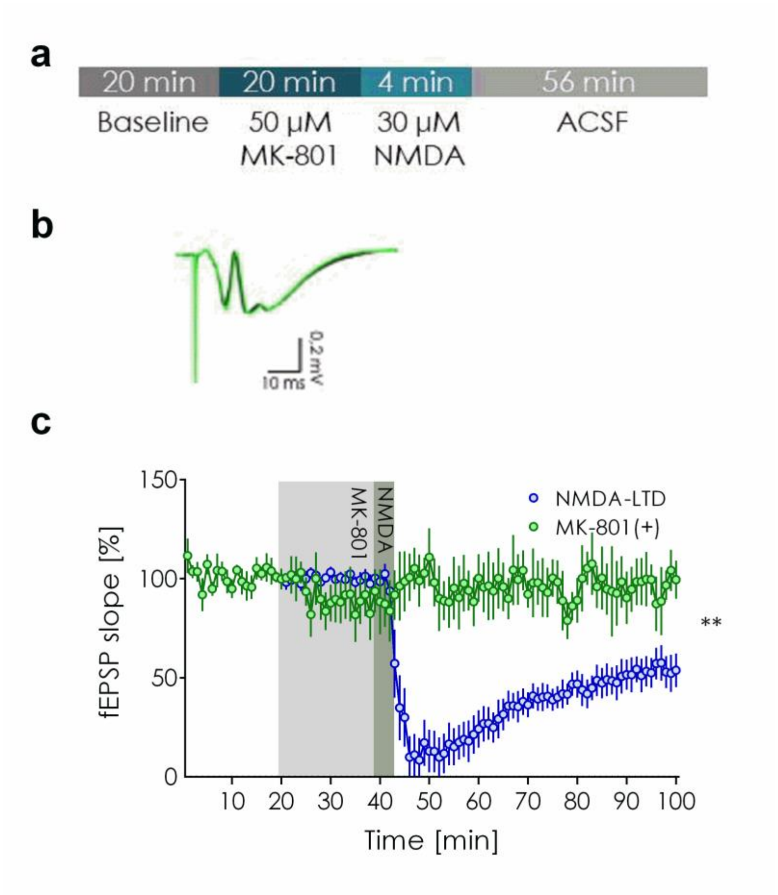

Supplementary Figure 1. NMDA-LTD involves synaptic NMDAR. (a) Experimental timeline. **(b)** Exemplary traces of fEPSPs before (black) and after (green) NMDA-LTD induction after MK-801 administration. **(c)** Synaptic depression was not induced when MK-801 was administered prior to induction (slices: NMDA-LTD= 8, MK-801= 5; the mean value of fEPSP slope during last 30 min of recording was compared between groups; unpaired t-test; $t(9)=3.773$, $p=0.0044$).

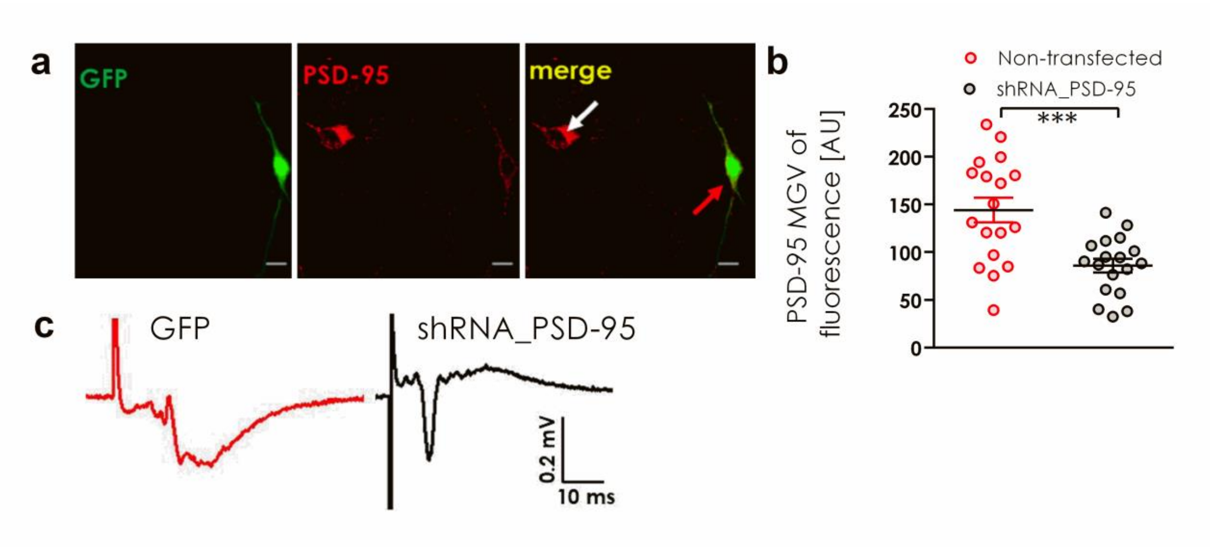

Supplementary Figure 2. Validation of shRNA for PSD-95. (**a**) Exemplary confocal single scans of dissociated hippocampal cultures transfected with plasmid encoding shRNA:PSD-95_GFP. Scale bar is 10 μm. White arrow indicates a non-transfected cell whereas the red arrow points at a transfected cell. (**b**) The MGV of PSD-95 fluorescence in the transfected cells was significantly lower than in non-transfected cells from the same slides (cells: Transfected= 18, Non-transfected= 18; unpaired t-test, $t(34)=3.923$, $p=0.0004$). (**c**) Exemplary traces of fEPSPs recorded in OHCs transduced with lentiviruses encoding GFP or shRNA:PSD-95. In shRNA expressing slices, no fEPSPs were found (n=10).
